# Role of water in complexation of 1,4,7,10,13,16-hexaoxacyclooctadecane (18-crown-6) with Li^+^ and K^+^ in hydrophobic 1-ethyl-3-methylimidazolium bis(trifluoromethanesulfonyl)amide ionic liquid

**DOI:** 10.1007/s10847-014-0427-1

**Published:** 2014-06-10

**Authors:** Tatsuya Umecky, Toshiyuki Takamuku, Ryo Kanzaki, Masaya Takagi, Eiji Kawai, Tomoya Matsumoto, Toshitaka Funazukuri

**Affiliations:** 1 Department of Chemistry and Applied Chemistry, Graduate School of Science and Engineering, Saga University, Honjo-machi, Saga, 840-8502 Japan; 2 Graduate School of Science and Engineering, Kagoshima University, 1-21-35 Korimoto, Kagoshima, 890-0065 Japan; 3 Department of Applied Chemistry, Faculty of Science and Engineering, Chuo University, 1-13-27 Kasuga, Bunkyo-ku, Tokyo 112-8551 Japan

**Keywords:** NMR, Ionic liquid, 18-crown-6, Complexation, Water

## Abstract

Complexation characteristics of 1,4,7,10,13,16-hexaoxacyclooctadecane (18-crown-6, 18C6) with Li^+^ and K^+^ in a hydrophobic ionic liquid of 1-ethyl-3-methylimidazolium bis(trifluoromethanesulfonyl)amide under dry and humid conditions at 298.2 K were studied by ^1^H and ^13^C NMR chemical shifts. The comparison of the ^1^H and ^13^C chemical shifts of 18C6 molecule between the dry and humid IL solutions without the alkali metal ions showed that uncomplexed 18C6 molecules are solvated by water molecules in the humid ionic liquid solution. The changes in the ^1^H and ^13^C chemical shifts of 18C6 ligand molecule with the increases in the Li^+^ and K^+^ concentrations revealed that in both dry and humid ionic liquid solutions 18C6 molecule forms 1:1 complexes with Li^+^ and K^+^. The ^1^H NMR data of water molecules in the humid ionic liquid solutions demonstrated that water molecules interact with Li^+^-18C6 complexes and free Li^+^, but do not with K^+^-18C6 complexes and free K^+^. The mechanisms of the formation of the Li^+^ and K^+^ complexes in the humid ionic liquid solution are different from each other due to the differences in the complex-water interactions.

## Introduction

**Fig. 1:**
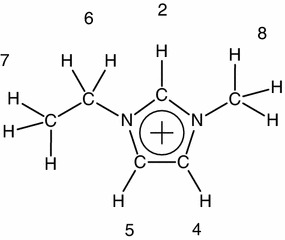
Molecular structure of [C_2_mim]^+^ with the notation of the hydrogen and carbon atoms

Ionic liquids (ILs) have come to attract attention as alternatives to volatile organic solvents for solvent extraction because they have several excellent properties, e.g., low volatility, low flammability, and high thermal and chemical stabilities [1]. Crown ether molecules are one of extracting agents to effectively transfer alkali and alkaline-earth metal ions from aqueous phase to organic liquid phase [2]. A number of fundamental data on the complexation of crown ethers with ionic species and neutral molecules are available in the literature [3]. Also, there are many reports on the complexation between metal ions and crown ether molecules in hydrophobic ILs from an engineering standpoint, as reviewed [1]. Water is the key to govern the complexation because a trace of water in ILs drastically changes the physical and electrochemical properties of the entire system [4–8]. Dietz and co-workers have determined the structure of the complex of dicyclohexyl-18-crown-6 (DC18C6) with Sr^2+^ transferred from aqueous solution to hydrophobic 1-alkyl-3-methylimidazolium bis(trifluoromethanesulfonyl)amide solution by EXAFS technique [9]. They have elucidated that each of two water molecules exists in the perpendicular direction of the DC18C6 plane of the Sr^2+^-DC18C6 complex. So, the effect of a trace of water in hydrophobic ILs cannot be disregarded in understanding the mechanism of the complexation of crown ether with metal ions. To clarify what role water molecules play in the complexation between crown ether and metal ions in hydrophobic ILs, it is essential to make clear the complexation in hydrophobic IL system with and without water. Although a molecular dynamics simulation has given the effect of water in hydrophobic ILs on the complex structure and the binding affinities of 1,4,7,10,13,16-hexaoxacyclooctadecane (18-crown-6, 18C6) [10], any experimental evidence has not yet been reported. Hence, we tried to elucidate the effect of water on the complexation of 18C6 with Li^+^ and K^+^ in 1-ethyl-3-methylimidazolium bis(trifluoromethanesulfonyl)amide ([C_2_mim][TFSA]) solutions at 298.2 K under both dry and humid conditions using ^1^H and ^13^C NMR techniques. The structure of C_2_mim^+^ with the notation of the H and C atoms is illustrated in Fig. 1.

## Experimental

### Chemicals

The IL used in this study was prepared and purified in our laboratory according to the same procedures as those in the previous report [11]. 18C6 (99 %) was purchased from Sigma-Aldrich Co. and used without further purification. Two alkali metal salts of lithium bis(trifluoromethanesulfonyl)amide (LiTFSA, 99.95 %) and potassium bis(trifluoromethanesulfonyl)amide (KTFSA, 99.8 %) were obtained from Sigma-Aldrich Co. and Kanto Chemical Co., respectively. The two alkali metal salts were dried for more than 24 h under reduced pressure at ~353 K before solution preparations. Deuterium oxide (99.9 D atom%) as a lock solvent and sodium 3-(trimethylsilyl)-1-propanesulfonate (DSS, 97 %) as an external reference substance were purchased from Sigma-Aldrich Co. and used without further purification.

### Sample preparation

The IL solvent under the dry condition was obtained by heating at ~333 K under reduced pressure for more than 72 h. The water content determined by a Karl-Fisher titration was 384 ppm. 18C6 was diluted with the IL solvent to reach the 18C6 concentration of 0.139 mol kg^−1^, corresponding to ~0.200 mol dm^−3^. Each of the alkali metal salts (LiTFSA and KTFSA) was also dissolved into the dry IL solvent at the metal ion concentration of 0.137 mol kg^−1^, corresponding to ~0.200 mol dm^−3^. The dry IL solutions of the alkali metal ions were mixed with the dry 18C6-IL solution at desired molar ratios of the alkali metal ion to 18C6. The dry IL solutions including both 18C6 and the alkali metal ion were diluted with the dry IL solvent again. The final concentrations were 0.0133 mol kg^−1^ for 18C6, up to 0.0403 mol kg^−1^ for the alkali metal ions, and 0.0213 mol kg^−1^ for water. Water and neat IL were agitated for 24 h at room temperature. The humid IL solvent was then obtained by separating the IL phase from two phase mixture of water and IL after standing for 24 h at ~333 K. The humid IL solvent had the water content of 2.09 × 10^4^ ppm. The humid IL solutions at various molar rations of the alkali metal ions to 18C6 were prepared in a similar manner to the preparation of the dry IL solutions. The final concentrations of 18C6, the alkali metal ions, and water in the humid IL solutions were 0.0134 mol kg^−1^, up to 0.0404, and 1.16 mol kg^−1^, respectively. To avoid the change in water concentration with passage of time, immediately after the sample preparations, a small portion of each of the IL solutions was expeditiously sealed into an NMR inner tube (Shigemi, SC-002).

### NMR measurement

A double tube constructed by a pair of the inner tube sealed with the IL solution and an outer tube filled with deuterium oxide solution including 1 wt% DSS was held for 30 min or more at 298.2 ± 0.1 K in a superconducting magnet. ^1^H and ^13^C NMR spectra were obtained with a JEOL ECA-500 spectrometer, where ^1^H and ^13^C resonance frequencies were 500.2 and 125.8 MHz, respectively. The digital resolutions of ^1^H and ^13^C NMR spectra were 0.14 and 0.48 Hz. The chemical shifts of ^1^H and ^13^C peaks of the IL sample solutions were determined using the peak of DSS as an external reference. The magnetic susceptibility correction for the dry IL systems has little effect on the ^1^H and ^13^C chemical shifts because the concentrations of 18C6, the alkali metal ions, and water are considerably lower than that of [C_2_mim][TFSA] (~2.5 mol kg^−1^). For the humid IL systems, in addition to the lower concentrations of 18C6 and the alkali metal ions, the volume susceptibility (−0.701 × 10^−6^ at 298.2 K) of the [C_2_mim][TFSA]-water binary mixture, which is estimated from the reported values of molar susceptibility, density, and molar mass of pure [C_2_mim][TFSA] and water [11, 12], is almost equivalent to the volume susceptibility of pure [C_2_mim][TFSA] (−0.700 × 10^−6^ at 298.2 K) measured by applying the external double reference technique [13] within ±0.1 %. It can be considered that there is no effect of water dissolved into the [C_2_mim][TFSA] on the volume susceptibilities of the sample solutions. For these reasons, we did not make magnetic susceptibility correction to the observed ^1^H and ^13^C data in this work.

### Formation constants (*K*_f_)

*K*_f_ values can be obtained from the chemical shifts of 18C6 as a function of molar ratio of the alkali metal ion to 18C6 ([M]/[18C6]) [14]. Briefly, in the case that 18C6 molecule forms only 1:1 complex with M^+^ ([M(18C6)]^+^), the 18C6 molecule was in equilibrium between complexed and uncomplexed forms.

1 $${\text{M}}^{ + } + 1 8 {\text{C6}} \rightleftharpoons \left[ {{\text{M}}\left( { 1 8 {\text{C6}}} \right)} \right]^{ + }$$

When the exchange between complexed and uncomplexed 18C6 is faster than NMR timescale, the observed NMR peak (*δ*_obs_) of 18C6 can be expressed by using the chemical shifts of uncomplexed (*δ*_L_) and complexed forms (*δ*_ML_) as follows:

2 $$\delta_{\text{obs}} \; = \;x_{\text{L}} \,\delta_{\text{L}} \; + \;x_{\text{ML}} \,\delta_{\text{ML}}$$

where *x*_L_ and *x*_ML_ are the mole fractions of uncomplexed and complexed 18C6, respectively. The mole fractions *x*_L_ and *x*_ML_ are given by

3 $$x_{\text{L}} \; = \;\frac{{C_{\text{L}} \; - \;[{\text{ML}}]}}{{C_{\text{L}} }}$$

and

4 $$x_{\text{ML}} \; = \;1\; - \;x_{\text{L}}$$

Here, [ML] is the concentration of 1:1 M^+^-18C6 complex. *C*_L_ is the total concentration of 18C6. In 1:1 M^+^-18C6 complexation reaction, the formation constant (*K*_f_) is defined by

5 $$K_{\text{f}} \; = \;\frac{{[{\text{ML}}]}}{{\left( {C_{\text{L}} \; - \;[{\text{ML}}]} \right)\;\left( {C_{\text{M}} \; - \;[{\text{ML}}]} \right)}}$$

where *C*_M_ is the total concentration of alkali metal ion. If Eq. (5) is rearranged using Eqs, (2)–(4),

6 $$\delta_{\text{obs}} \; = \;\delta_{\text{ML}} + \;\frac{{\left( {\delta_{\text{L}} \; - \;\delta_{\text{ML}} } \right)}}{{2\;K_{\text{f}} \;C_{\text{L}} }}\;\left\{ {\left( {K_{\text{f}} \;C_{\text{L}} \; - \;K_{\text{f}} \;C_{\text{M}} \; - \;1} \right)\; + \;\sqrt {\left( {K_{\text{f}} \;C_{\text{L}} \; - \;K_{\text{f}} \;C_{\text{M}} \; - \;1} \right)^{2} \; + \;4\;K_{\text{f}} \;C_{\text{L}} } } \right\}$$

In this study, the *K*_f_ values of 1:1 M^+^-18C6 complexes were determined from the change in ^13^C chemical shift of 18C6 with the molar ratio of the alkali metal ion to 18C6 ([M]/[18C6]).

## Results and discussion

**Fig. 2:**
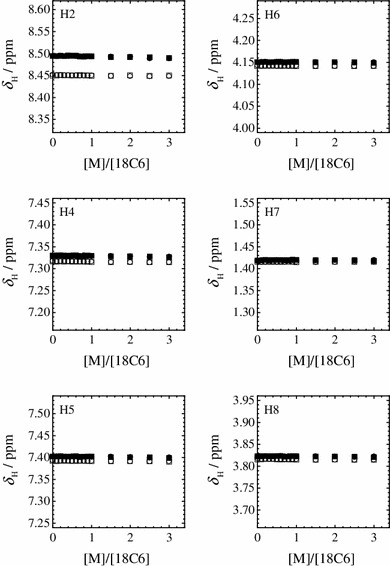
^1^H chemical shifts (*δ*_H_) of H2, H4, H5, H6, H7, and H8 of C_2_mim^+^ with LiTFSA (*circles*) and KTFSA (*squares*) in the dry (*open symbols*) and humid IL solutions (*closed symbols*) at 298.2 K as a function of the molar ratio of the alkali metal ion to 18C6 ([M]/[18C6])

**Fig. 3:**
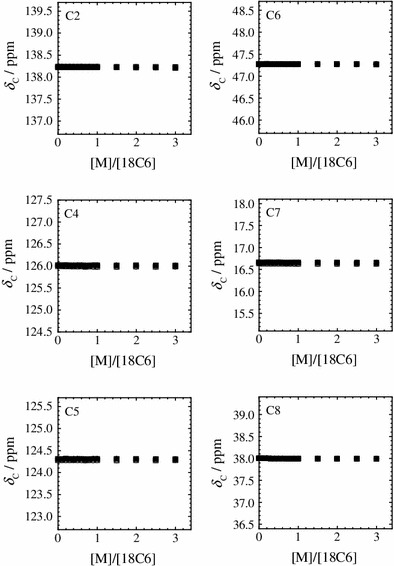
^13^C chemical shifts (*δ*_C_) of C2, C4, C5, C6, C7, and C8 of C_2_mim^+^ with LiTFSA (*circles*) and KTFSA (*squares*) in the dry (*open symbols*) and humid IL solutions (*closed symbols*) at 298.2 K as a function of the molar ratio of the alkali metal ion to 18C6 ([[M]/[18C6])

Figures 2 and 3 show the observed ^1^H and ^13^C chemical shifts (*δ*_H_ and *δ*_C_) of C_2_mim^+^ in the dry and humid IL solutions as a function of the molar ratio ([M]/[18C6]) of the alkali metal ion to the 18C6 ligand, respectively. The water contents in the dry and humid IL solutions are 384 and 2.09 × 10^4^ ppm, respectively. Without the alkali metal ions ([M]/[18C6] = 0), the *δ*_H_ values of C_2_mim^+^ are remarkably affected when the trace of water dissolves into the IL solutions, whereas the *δ*_C_ values of C_2_mim^+^ are unchanged against the water content. The effect of water on the H2 atom is more pronounced among all of the H atoms within C_2_mim^+^. This shows that the H2 atom of C_2_mim^+^ preferentially interacts with water molecule in the 18C6-IL solutions. The *δ*_H_ and *δ*_C_ values for all the H and C atoms of C_2_mim^+^ do not change against the increase in the concentration of the alkali metal ion. Thus, the differences of the *δ*_H_ and *δ*_C_ values between the dry and humid IL solutions are maintained constant in the [M]/[18C6] range studied. These results show that the C_2_mim^+^ does not significantly influence the complex formation of 18C6 with the alkali metal ions in the IL solutions.

**Fig. 4:**
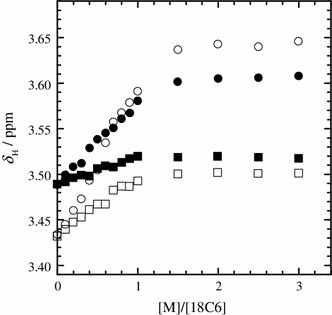
^1^H chemical shifts (*δ*_H_) of 18C6 in the dry (*open symbols*) and humid IL solutions (*closed symbols*) with LiTFSA (*circles*) and KTFSA (*squares*) at 298.2 K as a function of the molar ratio of the alkali metal ion to 18C6 ([M]/[18C6])

**Fig. 5:**
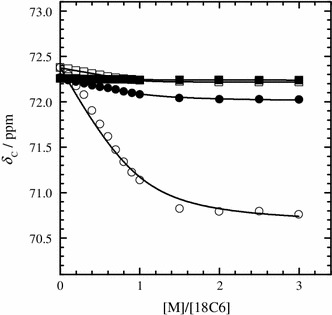
^13^C chemical shifts (*δ*_C_) of 18C6 in the dry (*open symbols*) and humid IL solutions (*closed symbols*) with LiTFSA (*circles*) and KTFSA (*squares*) at 298.2 K as a function of the molar ratio of the alkali metal ion to 18C6 ([M]/[18C6]). The solid lines represent the theoretical values obtained by a least-square regression with eq 6

The *δ*_H_ and *δ*_C_ values of 18C6 in the dry and humid IL solutions as a function of [M]/[18C6] are given in Figs. 4 and 5, respectively. At [M]/[18C6] = 0, the *δ*_H_ and *δ*_C_ values (*δ*_H_ = ~3.49 ppm, *δ*_C_ = ~72.3 ppm) of the 18C6 molecules without metal ion in the humid IL system are clearly different from those (*δ*_H_ = ~3.43 ppm, *δ*_C_ = ~72.4 ppm) in the dry one beyond the digital resolutions. This is attributed to the difference in the solvation structures of the uncomplexed 18C6 molecules between the dry and humid IL solutions. We have recently reported that water molecules in the humid IL solutions construct water-rich domains by hydrogen bonding among them [11]. In other words, IL-rich domains may simultaneously form in the humid IL solutions. The uncomplexed 18C6 molecules in the humid IL systems may be accommodated in the water-rich domains. In contrast, 18C6 molecules are solvated by C_2_mim^+^ and/or TFSA^−^ in the dry IL solutions. When the concentration of the alkali metal ion increases up to [M]/[18C6] = 1, the *δ*_H_ values of 18C6 molecules in the dry and humid IL solutions markedly increase. The values then reach a plateau above [M]/[18C6] = 1. The [M]/[18C6] dependence of the *δ*_C_ values is analogous to those of the *δ*_H_ values, despite the opposite direction. The break points at [M]/[18C6] = 1 in the plots of the *δ*_H_ and *δ*_C_ values clearly show that 18C6 molecule forms the 1:1 complex with Li^+^ and K^+^ in both of the dry and humid IL solutions.

On the assumption that 18C6 molecules are in an equilibrium between only the uncomplexed and the 1:1 metal-complexed forms, we attempted to determine the formation constants (*K*_f_) of the Li^+^- and K^+^-18C6 complexes in the IL solutions through Eq. (6). The ^13^C nuclei within 18C6 molecule are more sensitive to the formation of the 18C6 complexes with the alkali metal ions than the ^1^H nuclei because the C atoms bound to the ether oxygen atoms as the coordination sites for a metal ion. The *δ*_L_, *δ*_ML_, and *K*_f_ values for the four IL systems determined by the least-square regression on the *δ*_C_ data of the 18C6 molecules with Eq. (6) are summarized in Table 1. To our knowledge, there are a few literature data of log*K*_f_ of the Li^+^-18C6 complex in IL systems: 1.8 in 1-ethyl-3-methylimidazolium chloride with 55 mol% aluminium(III) chloride [15] and 2.0 in 1-butyl-3-methylimidazolium bis(trifluoromethanesulfonyl)amide [16]. Judging from the *K*_f_ values of the Li^+^-18C6 complexes in the literature, the present values for the [C_2_mim][TFSA] systems are reasonable. On the other hand, the *K*_f_ value of the K^+^-18C6 complex in ILs is not available. In both dry and humid IL solutions, the *K*_f_ values of the K^+^-18C6 complex are larger compared with those for the Li^+^-18C6 ones. This arises from the higher size fitness of K^+^ (ionic radius = 0.137–0.164 nm [12] ) than Li^+^ (ionic radius = 0.059–0.092 nm [12] ) to the 18C6 cavity (cavity radius = 0.130 nm [17] ). The important finding in the present results is that water molecules dissolved in the IL solutions hinder the formation of the Li^+^- and K^+^-18C6 complexes. The inhabitation of the alkali metal-18C6 complex formation on adding water is ascribed to the strong hydration of the uncomplexed 18C6 molecules by water molecules in the humid IL solutions.

**Table 1:** Chemical shifts of the uncomplexed (*δ*_L_) and the alkali metal-complexed 18C6 molecules (*δ*_ML_), and the formation constants (*K*_f_) of the Li^+^-18C6 and K^+^-18C6 complexes in the dry and humid IL solutions at 298.2 K determined from the *δ*_C_ data using Eq. (6)

Solvents	Alkali metal ions	*δ*_L_ (ppm)	*δ*_ML_ (ppm)	log (*K*_f_) (mol^−1^ dm^3^)	AD%^a^
[C_2_mm][TFSA] (dry)	Li^+^	72.4	70.6	2.6	0.05 (0.15)
	K^+^	72.4	72.2	3.6	0.00 (0.01)
[C_2_mm][TFSA] (humid)	Li^+^	72.2	72.0	2.5	0.01 (0.02)
	K^+^	72.3	72.2	2.6	0.00 (0.00)

^a^Absolute deviation AD % is given by |1 − *δ*_prd_/*δ*_exp_| × 100, where *δ*_prd_ and *δ*_exp_ are the predicted and experimental chemical shifts, respectively. The maximum of AD% are given in parentheses

Focusing on the *δ*_L_ values of the uncomplexed 18C6 molecule in Table 1, for both dry and humid IL solutions, the values of the Li^+^- and K^+^-18C6 systems are in agreement with each other. However, a comparison between the *δ*_L_ values for each metal system in the dry and humid IL solutions shows a slight difference, e.g., *δ*_L_ = 72.4 and 72.2 ppm for the Li^+^ system, respectively. This comes of the difference in the solvation structures of the uncomplexed 18C6 molecules between the dry and humid IL solutions. 18C6 molecules in the humid IL solutions are solvated by water molecules, as described above. In the dry IL solutions, they are solvated by C_2_mim^+^ and/or TFSA^−^.

On the other hand, the *δ*_ML_ values of the K^+^-18C6 complex in the dry and humid IL solutions are almost equal to each other. This indicates that the structure of the K^+^-18C6 complex is the same in both the dry and humid IL solutions. Hence, water molecules in the IL solutions may not contribute to the structure of the 1:1 K^+^-18C6 complex. However, the significantly different *K*_f_ values of the K^+^-18C6 complex in the dry and humid IL solutions suggest that water molecules influence the mechanism of the complex formation. In the humid IL solution, the K^+^-18C6 complex might move to the IL-rich domains after its complexation in the water-rich domains because of its hydrophobicity. Unlike in the case of the K^+^-18C6 system, the *δ*_ML_ value of the Li^+^-18C6 complex under the humid condition is quite distinct from that under the dry condition. The *δ*_ML_ value of the Li^+^-18C6 complex in the humid IL solution is fairly larger compared to that in the dry IL solution. This shows the different structure of the Li^+^-18C6 complex in the dry and humid IL solutions. The smaller *δ*_ML_ of the ^13^C nuclei for the dry IL solution means the higher coordination state of 18C6 molecule. In contrast, the coordinating ability of Li^+^ to the 18C6 ligand molecule under the humid condition is lower than that under the dry one. Our previous findings showed that Li^+^ more preferentially interact with water molecules than with C_2_mim^+^ and TFSA^−^ in the humid [C_2_mim][TFSA] solutions [11]. Water molecules in the humid 18C6-IL solution may also interact with both Li^+^ isolated from the 18C6 ligand and incorporated into the ligand. The Li^+^-water interactions may weaken the Li^+^-18C6 interactions in the humid IL system.

**Fig. 6:**
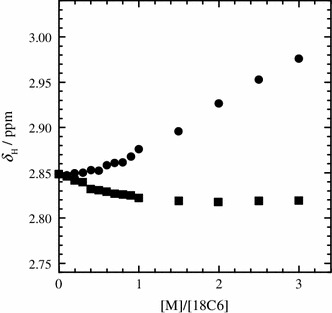
^1^H chemical shifts (*δ*_H_) of water in the humid IL solutions with LiTFSA (*circles*) and KTFSA (*squares*) at 298.2 K as a function of the molar ratio of the alkali metal ion to 18C6 ([M]/[18C6])

Figure 6 shows the *δ*_H_ values of water molecules in the humid IL solutions as a function of [M]/[18C6]. Unfortunately, the *δ*_H_ of the trace of water in the dry IL solutions was not able to be determined due to its low concentration. With the increase in the [M]/[18C6] ratio of K^+^ to 18C6 ligand, the *δ*_H_ value of water molecules in the humid IL solution decreases and then remains unchanged. This change in the *δ*_H_ values for the water molecules with increasing K^+^ concentration is strikingly similar to those for *δ*_C_ of the 18C6 molecules (Fig. 5). In Fig. 6, the largest *δ*_H_ value for the K^+^ system at [M]/[18C6] = 0 in the ratio range examined is the definite evidence of the hydration of the 18C6 ligand. Below [M]/[18C6] = 1, the water-18C6 interactions become weaker by the enhancement of the K^+^-18C6 interactions. In other words, the decrease in the *δ*_H_ values is due to the dehydration of 18C6 molecule during the complex formation of the 18C6 ligand with K^+^. The plateau of the *δ*_H_ values of water molecules above [M]/[18C6] = 1 signifies that water molecules in the IL solution hardly interact with the excess of the uncomplexed K^+^ ions. Water molecules in the IL solutions more strongly interacts with the uncomplexed 18C6 molecules than the K^+^-18C6 complexes and K^+^ isolated. On the other hand, as the formation of the Li^+^-18C6 complex proceeds in the humid IL solution, the gradual increase in the *δ*_H_ values of water molecules is followed by the drastic increase. The gradual increase in the *δ*_H_ values below [M]/[18C6] = 1 is most commonly due to the interactions between water molecules and the complexed Li^+^. The drastic increase in the *δ*_H_ values above [M]/[18C6] = 1 results from the strong interactions of water molecules with the excess Li^+^. The [M]/[18C6] dependence of the *δ*_H_ values of water molecules for the Li^+^ system reveals that water molecules more weakly interact with the complexed Li^+^ than the uncomplexed Li^+^. Therefore, water molecules more strongly interact with the species in the Li^+^ systems in the order of free 18C6 < Li^+^-18C6 complex < free Li^+^. This result for the Li^+^ system is completely different from the K^+^ system (K^+^-18C6 complex ≈ free K^+^ < free 18C6). Considering that water molecules interact with the Li^+^-18C6 complex as well as the uncomplexed 18C6 molecules, the formation of the Li^+^-18C6 complex is taken place in the water-rich domains in the humid IL solution. The Li^+^-18C6 complexes may remain in the water-rich domains.

## Conclusion

18C6 molecules are solvated by water molecules in the water-rich domains in the humid IL solutions. In the humid IL solutions, the 18C6 molecules form the alkali metal ions-18C6 complexes at the stoichiometric ratio of the unity for both Li^+^ and K^+^ systems. However, the formation mechanisms of the Li^+^- and K^+^-18C6 complexes are different from each other due to the difference in the interactions of water molecules with the complexes. The Li^+^-18C6 complexes form in the water-rich domains and steady in the water-rich domains by the relatively strong interaction between water molecules and the Li^+^-18C6 complex. On the other hand, the K^+^-18C6 complexes formed in the water-rich domains may move into the IL-rich domains owing to very weak interactions between water molecules and the complex.
